# Effects of Different Freezing Methods on Physicochemical Properties of Sweet Corn during Storage

**DOI:** 10.3390/ijms24010389

**Published:** 2022-12-26

**Authors:** Mingying Wang, Siyuan Jin, Zhaoyang Ding, Jing Xie

**Affiliations:** College of Food Science and Technology, Shanghai Ocean University, Shanghai 201306, China

**Keywords:** sweet corn, freezing methods, antioxidant enzymes, preservation, shelf life

## Abstract

Fresh sweet corn has a series of physiological and biochemical reactions after picking due to the high moisture content, leading to damaged nutritional value. Rapid freezing of sweet corn after harvest can minimize tissue damage and quality deterioration. In this study, freshly harvested sweet corn was frozen by ultrasound-assisted freezing, brine freezing, strong wind freezing, and refrigerator freezing. The effects of different freezing methods on hardness, water loss, color, epidermal structure, soluble solids content, soluble sugars content, peroxidase (POD), catalase (CAT), and ascorbate peroxidase (APX) activities of frozen sweet corn during storage were investigated. The results showed that brine freezing and strong wind freezing could effectively reduce the quality loss of sweet corn, keep the color, soluble sugars, and soluble solids content of the sweet corn, delay the decrease in antioxidant enzyme activity, and maintain the quality of sweet corn during long term storage.

## 1. Introduction

Sweet corn has light yellow grains and a sweet taste, with a crunchy texture, high moisture, and sugar content. It is also rich in vitamins, amino acids, minerals, and trace elements, as well as nutrients such as glutathione and linoleic acid [1]. Sweet corn breathes vigorously after picking up due to its high moisture and sugar content, which results in a serious loss of nutrients, declining freshness, tenderness and sweetness, rough taste, and breeding of pathogenic microorganisms. It seriously affects the edible quality and taste of sweet corn and shortens the storage period and shelf life [2,3].

The postharvest management of sweet corn is critical to reduce product waste that helps maintain product quality throughout the entire supply chain [3]. Temperature is an important factor affecting the storage and preservation of sweet corn; as the temperature rises, the respiratory intensity increases and sweet corn metabolism increases, accelerating maturation and aging, shortening the shelf life, resulting in the loss of economic value and food value [4]. As sweet corn respirates vigorously, cell metabolism increases, resulting in sweet corn moisture loss wilting, withering bracts green, wilting, and seed nutrient content decrease. Therefore, it is necessary to reduce the respiration intensity of sweet corn during storage [5].

Freezing is considered a processing method to stop or slow down biochemical reactions in fruit and vegetable preservation [6]. It can quickly remove the field heat carried by sweet corn itself after harvesting and inhibit respiration, thus, slowing down the speed of sweet corn maturation and aging [7]. This involves reducing the temperature of the product, usually to −18 °C or lower. At temperatures below −10 °C, little microbial growth is possible, the rate of chemical reactions is greatly reduced, and the metabolic reactions of cells are delayed [8,9]. In recent years, novel methods have been explored to improve and control the crystallization process, of which ultrasonic crystallization has proved to be extremely useful as it improves the nucleation and crystal growth rates [10].

Refrigeration is one of the common preservation methods of food, which can make the refrigerator cool to the ideal ambient temperature faster and shorten the time for the fruit to reach the ideal temperature [11]. The application of ultrasound in the freezing process helps to inactivate enzymes and microorganisms [12,13,14]. Compared with conventional freezing, the advantages of ultrasound-assisted freezing include a high freezing rate, fast crystallization rate, uniform ice crystal distribution, and better microstructure of cells [15]. Xin et al. [16] investigated the freezing characteristics of broccoli during ultrasound-assisted freezing and showed that changes in weight loss, color, firmness, and L-ascorbic acid content of ultrasound-assisted frozen broccoli were significantly suppressed during the freezing process. Frozen brine preservation refers to a frozen preservation technology that puts the material to be frozen directly into the cold brine for direct contact heat exchange. Brine freezing preservation technology is widely used in the freezing preservation process of carrots, peanuts, fruits, potatoes, etc. The freezing preservation of aquatic products on ocean ships also widely adopts the cold brine freezing technology. Lucas et al. [17] studied the changes in quality indicators, such as salt absorption of apple slices during cold brine impregnation freezing and storage. Caletto et al. [18] investigated the quality changes of strawberries during brine freezing. The results showed that compared with the control group, the loss of juice after thawing was smaller after brine freezing. During strong wind freezing, fans force cold air to flow through the trays at high speeds, promoting faster cooling than room cooling. Strong wind freezing is the most common method used to extend the shelf life of perishable foods [19].

In this study, four different freezing methods were used to treat fresh sweet corn, and these four more energy-efficient and environmentally friendly freezing methods were compared. In addition, this study was conducted by comparing the effects of four freezing methods on the physical and chemical properties of sweet corn to select better the best freezing method for reducing post-harvest losses.

## 2. Results

### 2.1. Color Difference and Sensory Analysis of Sweet Corn

**Figure 1:**
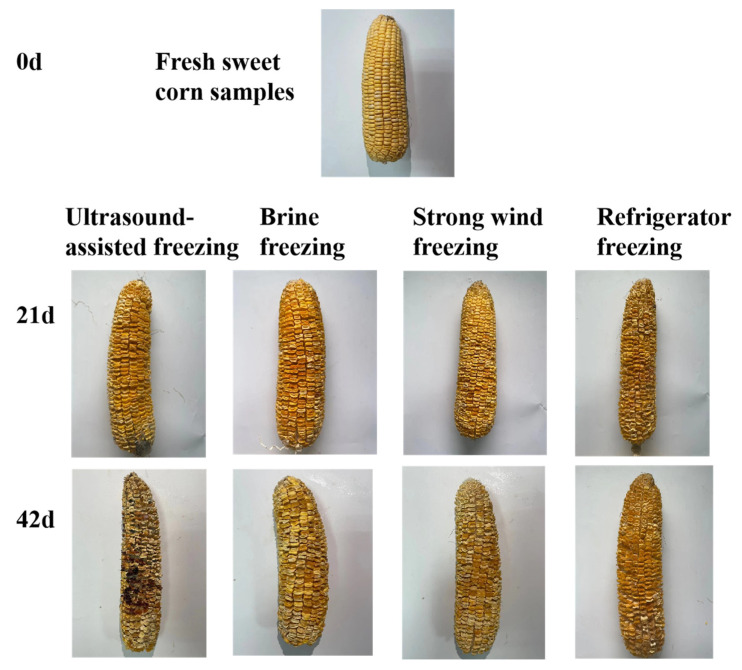
The appearance changes of sweet corn during storage with four different freezing methods.

**Figure 2:**
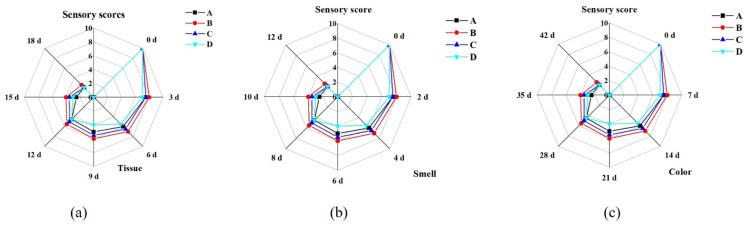
Sensory changes in tissue (**a**), smell (**b**), and appearance (**c**) of sweet corn during storage by four different freezing methods. (A: Ultrasound-assisted freezing, B: Brine freezing, C: Strong wind freezing, D: Refrigerator freezing).

The sensory quality of frozen sweet corn varied with storage time, as shown in Figure 1. From Figure 2, it can be seen that the treatment groups with brine freezing and strong wind freezing had the highest sensory scores in terms of sensory evaluation of both muscular tissue, odor, and appearance, while the treatment group with refrigerator freezing had the lowest scores.

Color is an important characteristic of fruits and vegetables, as it is an indicator of ripeness and freshness; it is related to the nutritional quality and sensory quality of sweet corn [20]. As shown in Table 1, the L* values of sweet corn showed a decreasing trend from day 21 onwards for different freezing treatments due to the darkening of the color caused by browning. Chrominance index a* indicates the degree of red and greenness of sweet corn. As shown in Table 1. The b* value represents the level of the yellow color of sweet corn, and a large value indicates a serious yellowing of sweet corn. As the freezing time increased and the temperature decreased, the refrigerator freezing treatment group had a color difference, as seen in Table 1, with significant differences compared to the other treatment groups. Among the other treatment groups, strong wind freezing was the best, probably because the strong wind treatment group would reduce the free water content in sweet corn and slows down the movement rate of crystal water, thus, effectively controlling the transpiration rate and biological activity.

### 2.2. The Epidermal Structure of Sweet Corn under Scanning Electron Microscope

**Figure 3:**
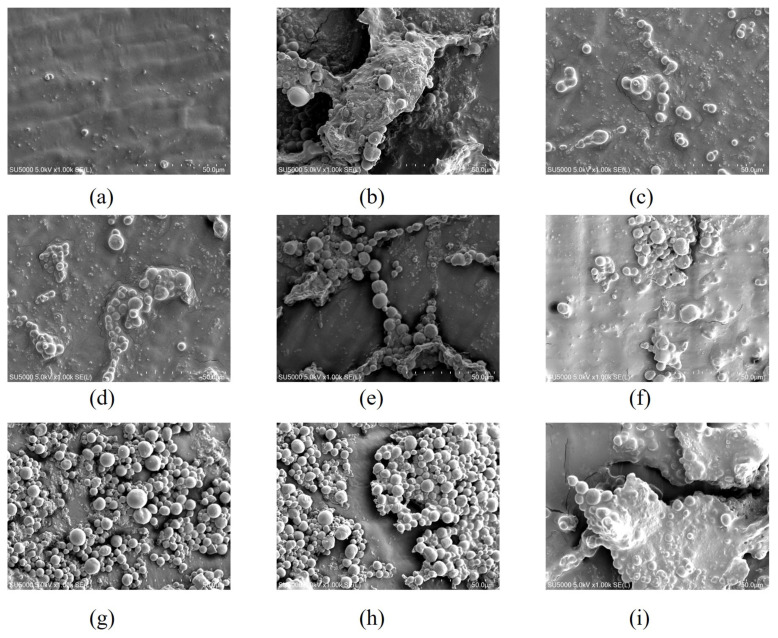
Scanning electron microscopy images of fresh sweet corn in the early storage period (**a**); ultrasound-assisted freezing of sweet corn epidermis in the middle storage period (**b**), brine freezing of sweet corn epidermis in the middle storage period (**c**), strong wind freezing of sweet corn epidermis in the middle storage period (**d**) and refrigerator freezing of sweet corn epidermis in the middle storage period (**e**); ultrasound-assisted freezing of sweet corn epidermis in the post storage period (**f**), brine freezing of sweet corn epidermis in the post storage period (**g**), strong wind freezing of sweet corn epidermis in the post storage period (**h**), and refrigerator freezing of sweet corn epidermis in the post storage period (**i**).

From Figure 3a, it can be seen that the initial sweet corn is structurally intact and neatly arranged, and in the middle stage of freezing, the sweet corn epidermis is relatively full and contains high water content, with almost unchanged cell morphology and high integrity of epidermal cells. As can be seen in Figure 3f–i, with the increase in freezing days, the cell structure was deformed and began to fold and curl, with a gradual increase in the cell gap as well as an obvious perforation of the cell wall. This indicates that the cellular integrity of sweet corn was damaged. The combination of cell morphology and pore opening showed that brine freezing (Figure 3c–g) gave the best results, followed by strong wind freezing (Figure 3d–h).

### 2.3. Analysis of Hardness, Water Loss, Soluble Sugars, and Soluble Solids Content of Sweet Corn

Hardness also determines the consumer’s acceptance of sweet corn. Frozen sweet corn was tender with an initial hardness of 1874.14 g. As storage time increased, the water content of the kernels decreased, the skin layer became thicker, and the hardness of the kernels gradually increased. As shown in Table 2. The hardness of all sweet corn samples showed a trend of decreasing and then increasing during storage, which may be due to the freezing process reducing syneresis and reducing the hardness of the corn by reducing the size of the ice cells [21]. The results of the study showed that the best results were obtained for sweet corn treated with strong wind freezing. This may be due to the reduced consumption of organic matter and water under strong wind to maintain the tenderness of the sweet corn.

The sweet corn freshness period, with the increase in storage time, produces different degrees of water loss phenomenon, so that sweet corn kernel shrinkage, the degree of freshness is reduced, which seriously affects its commercial value. Therefore, the weight loss rate is a very important index to detect the freshness of preserved fruits and vegetables. During storage, the weight loss rate gradually increased in the four treatment groups, with strong wind freezing and brine freezing being the most effective. The weight loss after 42 days of storage of strong-wind frozen sweet corn was only 34.10%, as shown in Table 2. The weight loss of ultrasound-assisted frozen sweet corn was as high as 39.87% after 42 days of storage.

The Initial soluble sugar content of sweet corn was 28.13 mg/g, as shown in Table 1. After 42 days of storage, soluble sugars decreased from 28.13 to 13.00, 12.44, 16.31, and 7.75 mg/g in samples stored in ultrasound-assisted freezing, brine freeze, strong wind, and refrigerator freeze, respectively.

It can be seen from Table 2 that the soluble solids content of sweet corn treated with different freezing methods showed an overall decreasing trend. Among them, the soluble solids content of the sweet corn treated with refrigerator freezing decreased the fastest compared to the other three groups, with only 6.10% soluble solids content at 42 days. In contrast, brine freezing and strong wind freezing had the best results, and the content of soluble solids decreased more slowly; at day 42, the content of soluble solids was 7.2% and 6.8% for strong wind freezing and brine freezing, respectively.

### 2.4. Antioxidant-Related Enzyme Analysis

**Figure 4:**
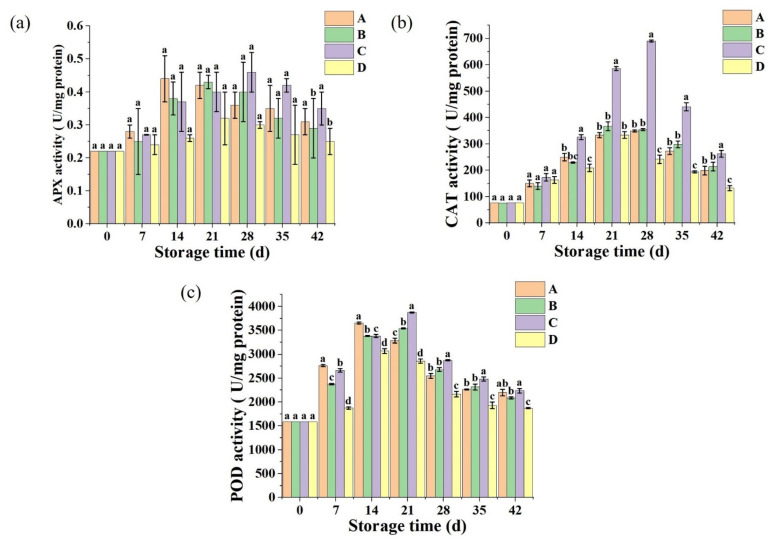
Treatment changes of APX (**a**), CAT (**b**), and POD (**c**) activities of sweet corn by different freezing methods. Values with different lowercase letters in the same sampling are significantly different (*p* < 0.05). (A: Ultrasound-assisted freezing, B: Brine freezing, C: Strong wind freezing, D: Refrigerator freezing).

Fruit and vegetable cells contain many enzymes, among which oxidoreductases are mainly POD, CAT, and PPO. If POD and PPO activity can be effectively inhibited during storage, it can avoid sweet corn browning and delay sweet corn ripening and aging. As shown in Figure 4a,b, the activities of APX and CAT of sweet corn treated with different freezing methods increased at the beginning of the storage period and peaked at day 28. At this time, the sweet corn treated with brine and strong wind freezing had the highest APX activity during the storage period with 0.40 and 0.46 U/mg protein, respectively, while the sweet corn treated with freezing in the refrigerator had the lowest APX activity. The CAT activities of sweet corn treated with brine freezing and strong wind freezing were 354 and 650 U/mg protein, respectively, during storage, after which the activities of APX and CAT started to decrease. At day 42, sweet corn frozen in the refrigerator exhibited relatively low CAT and APX activities of 132 and 0.24 U/mg protein, respectively, while sweet corn was frozen in brine and strong air exhibited higher APX and CAT activities.

As shown in Figure 4c, the POD activity of sweet corn treated with different freezing methods increased and then decreased during storage, peaking at 21 days for POD activity. In contrast, POD activity was highest in sweet corn treated with brine freezing and strong wind freezing, while it remained low in sweet corn treated with refrigerator freezing.

## 3. Discussion

The color parameter measured in sweet corn is an important factor influencing consumer preference [22]. The quality of sweet corn rapidly deteriorates after harvest, as evidenced by dryness, discoloration, moisture loss, loss of sweetness, and off-flavors [23]. As storage time increases, the maturity of sweet corn gradually increases, and its sensory quality gradually decreases. Due to the influence of freezing, sweet corn produces chilling injury and reduces the activity of internal antioxidant enzymes by gradually browning [24]. Browning is a phenomenon that is described in various vegetables such as lettuce or avocados such as sliced onions or apple juice [25,26]. Liang et al. [27] treated fresh litchi by freezing and soaking. The results showed that the mechanical damage of ice crystals on the pericarp would enhance enzyme oxidation. Because of the destruction of cell walls, the contact between phenols, oxygen, and enzymes increased, which may be the reason for the increased browning observed in quick-frozen litchi compared with fresh litchi. Enzymatic browning may be due to the degradation of polyphenols by enzymes such as polyphenol oxidase and peroxidase [28]. These degradation reactions are likely to be accelerated by membrane damage, resulting in the color change of sweet corn.

Weight loss is an important indicator of the postharvest storage quality of the fruit; the amount of moisture and organic matter content is an important influencing factor in determining the quality of frozen sweet corn. The reason for the increase in weight loss rate may be the loss of water and nutrients caused by transpiration, respiration, and a series of chemical reactions of sweet corn during storage. In the postharvest life cycle, dehydration may be caused by low moisture environments. Loss of 2% of seed moisture can lead to significant seed depression, which affects the appearance of freshness. Frozen sweet corn quality loss is influenced by different freezing treatments and storage times. Vegetables are subject to respiration and transpiration even after harvest, resulting in a continuous loss of quality. Due to the freezing effect, water evaporates, and, therefore, weight loss occurs during the freezing process [29].

The hardness of sweet corn is small at first because sweet corn is harvested at the ripening stage, with full kernels, high water content, and good elasticity. In addition, with the extension of the storage period of sweet corn after harvesting high respiratory intensity, and rapid changes in nutrient content, seeds lose water and harden. In the late storage period, the hardness continued to become greater. Throughout the preservation process, brine freezing treatment was the most effective, which also corresponded to the results of sweet corn water loss rate. This can be attributed to the fact that the brine treatment inhibited the volatilization of sweet corn water and slowed down the increase in hardness of frozen sweet corn.

Freezing can cause physical changes in some foods, which requires some new freezing processes to improve freezing conditions and product quality [30]. Combining freezing with other auxiliary methods can improve the quality of food by controlling the way ice forms in the food during freezing. Sweet corn epidermal cells contract and are compressed by surrounding ice crystals after freezing, and this compression often leads to cell damage and uneven cell distribution. The microstructure of sweet corn epidermal cells can be seen in the scanning electron microscope. Due to the faster freezing rate of brine freezing and strong wind freezing, there were fewer ice crystals in the cells and less damage to the sweet corn epidermal cells.

The soluble sugar content of frozen sweet corn is extremely important and depends on the variety, harvest time, and postharvest practices [31]. In this experiment, the highest content of soluble sugars was found in frozen sweet corn treated with strong wind freezing. During the experiment, strong wind freezing was the most effective. This reduction may be related to respiration and sugar conversion. Low temperatures usually induce the conversion of starch to reducing sugars [32].

The soluble solids content reflects the physiological state and quality of the plant [33]. The soluble solids content is one of the key indicators for judging the physiological quality of sweet corn. It can effectively reflect the changes in nutrients and postharvest maturity of sweet corn during storage [34]. The main reason for the decrease in soluble solids content was related to the respiration of sweet corn, which was energetic in its respiratory and metabolic activity, consuming a large amount of its own nutrients and decreasing soluble solids content [35]. At the end of storage, the soluble solids content of sweet corn frozen in brine and strong wind freezing decreased less, while the soluble solids content of refrigerator freezing decreased more. This may be caused by the brine freezing largely inhibited the respiration rate of sweet corn as well as microbial growth [6].

The oxidation reaction in sweet corn has been going on during freezing, and POD, SOD, and CAT in sweet corn are three enzymes that are closely related to the antioxidant reaction. The activities of these enzymes reflect the changes in the oxidative activity of sweet corn during freezing. ROS are continuously produced in different plant cell compartments as by-products of various metabolic pathways. In general, excess ROS may cause oxidative damage to cells and eventually induce fruit senescence [36]. Fortunately, plants possess effective enzymatic antioxidant defense systems, including the ability to scavenge ROS to protect plant cells from oxidative damage to SOD, CAT, and APX [37,38]. To reduce oxidative stress, plants have defense systems to scavenge excess ROS. Antioxidant enzyme systems, consisting of POD, CAT, and APX, are important systems to protect cells from damage, thereby maintaining intracellular ROS homeostasis, slowing membrane lipid peroxidation, and resisting stress. Thus, high levels of antioxidant enzymes are essential to mitigate oxidative damage [39].

In addition, APX, CAT, and POD can remove superoxide anion radicals generated during the physiological activity of sweet corn, thus, reducing the oxidative effect of free radicals on tissue cells [40,41]. Thus, these antioxidant enzymes could slow down the process of sweet corn peroxidation and maintain the integrity of its cell membrane structure. In this study, the use of both strong wind freezing and brine freezing effectively enhanced the activity of these three antioxidant enzymes and effectively extended the shelf life of sweet corn. The antioxidant enzyme activity of the brine-treated sweet corn was the best because salt not only reduces the water activity of the food to prevent spoilage but also changes the cellular osmotic pressure to detach the microbial protoplasm from the cell membrane, killing the microorganism, which is the cellular ability to scavenge free radicals is enhanced. Strong wind freezing by the fan to force the circulation of cold air flow, more balanced distribution of cold air, sweet corn freezing, and refrigeration effect is better.

## 4. Materials and Methods

### 4.1. Sweet Corn Materials

The common sweet corn was picked from the fields in the Fengxian district, Shanghai and transported to the lab within 1 h; then, the skin was peeled off, washed with distilled water, and the surface water dried with a paper towel. Sweet corn with similar size and maturity was selected for the experiment.

In this study, 84 sweet corn of similar size were randomly selected, washed, and dried, and then divided into four experimental groups on average and treated with four different methods. After 0, 7, 14, 21, 28, 35, and 42 days of storage, the sweet corn was removed from the cold storage and placed in a 25 °C constant temperature and humidity incubator (LHS-100CA, Shanghai Yiheng Instruments Co., Ltd., Shanghai, China). At 25 °C, the 20 seeds were randomly selected from the sweet corn in the experimental group to determine the physiological and biochemical indexes.

### 4.2. Freezing Methods

The following four kinds of freezing treatments were carried out:

(i) Ultrasound-assisted freezing: An ultrasonic-assisted freezer operating at 20 and 30 kHz with a power dissipation of 150 W (Euronda company, Vicenza, Italy) was used for 8 selected sweet corns for freezing (2 °C) in each treatment group.
(ii) Brine freezing: Each treatment group selected 8 fresh sweet corn to be stored in a −20 °C refrigerator (BCD-372 WTV, Qingdao, Chinese) saturated chlorine salt water soaking tank.
(iii) Strong wind freezing: The sweet corn was spread evenly on a shallow uncovered tray of 1.5–1.5 m at a distance of 20 cm and stored in a forced ventilation refrigerator (BCD-252MHV, SSEC, Suzhou, China) at 4 °C with a wind speed of 8 m/s.
(iv) Refrigerator freezing: The cleaned sweet corn of the same size is stored in a refrigerator (BCD-252MHV, SSEC, Suzhou, China) at −3 °C.

### 4.3. Appearance and Sensory Scores

The color evaluation was performed using a colorimeter (Minolta, Osaka, Japan, model-NH310). The L* value represents the brightness of sweet corn, with larger L* values indicating brighter color. The instrument was aligned with a black-and-white standard and observed experimental data for L*, a*, and b*, repeated three times, and the average value was taken.

Sensory quality analysis was recorded using characteristics based on: muscular tissue, smell, and appearance. The evaluation team consisted of 10 people with rich experience in the sensory evaluation of each sample reasonably during the experiment. The full score of the three indicators is 10. The specific evaluation criteria are shown in Table 3.

### 4.4. Determination of Physical Indicators

The hardness of fruits was measured using the FHM-5 Fruit Hardness Analyzer (Takemura Electric Industrial Co., Ltd., Tokyo, Japan). Following contact with sweet corn, the probe achieved a penetration rate of 20 mm/min within 10 mm. In each experiment, 5 seeds in each treatment were used for hardness measurement [35].

Sweet corn kernels were first homogenized and centrifuged at 15,000 rpm (Beckman J20–2) for 20 min. The supernatant was collected for analysis in subsequent experiments. The supernatant was tested with a digital display refractometer (PR-32a, Atago Co., Ltd., Japan). For each treatment group, soluble solids content was determined using three sweet corns.

The sweet corn was weighed at the beginning of the experiment using an electronic balance (Changshu Jinyang Balance Instrument Factory). Then the frozen corn sampled each time is weighed. The weight loss was calculated as the percentage reduction in the weight of sweet corn compared to the initial weight.

Measurements were performed according to Southgate’s method with modifications. Kernels were selected randomly from the central region of frozen sweet corn. The kernels were ground at 4 °C and 5 g of the ground sample was weighed into a 50 mL Falcon tube, extracted with two 25 mL portions of 50:50 ethanol: deionized water, and centrifuged for 20 min each time at 4 °C using an Eppendorf centrifuge (Hamburg, Germany); 2 mL of the supernatant was passed through a 0.2 µm water syringe filter and the sugar content was measured [42].

Remove the skin of the frozen sweet corn sample with tweezers, then place it on the short cross-section of the aluminum sample, coat the gold with double-sided adhesive tape, and coat the gold with ion sputtering. After 20 min, the sample was observed and captured under a scanning electron microscope (Hitachi, Japan).

### 4.5. Antioxidant Enzyme Assay

The activity of APX was measured using the method described in Javadi [43]: 1 mL of the assay was mixed from 100 μL 50 mM of ice-cold potassium phosphate (pH 7.0), 50 μL 10 mM H_2_O_2_, 50 μL 10 mM of ascorbic acid, 790 uL of distilled water and 10 uL of sweet corn extracts. Changes in absorption were monitored at 290 nm. The APX activity is reported as a U/mg protein.

CAT enzyme activity was performed according to Periyar Selvam with some modifications [44]. Analysis of the reaction mixture consisted of sodium phosphate buffer, H_2_O_2,_ and sweet corn extract (50 μL), after which hydrogen peroxide decomposition was observed at 240 nm (Multiskan GO microplate Spectrophotometer, Finland). Enzyme activity was reported as units per milligram of protein (U mg^−1^ protein). One unit represents the amount of catalase required to convert U/mg protein [45].

The activity of the POD was analyzed by following the method of Jiang with minor modifications reported by Periyar Selvam [44]. The sweet corn extracts were mixed with a buffer solution containing sodium phosphate solution and guaiacol and incubated at 30 °C for 5 min. Subsequently, H_2_O_2_ was added, and the increase in absorbance at 460 nm was measured for 120 s (Multiskan GO microplate Spectrophotometer, Finland). One unit of enzyme activity was expressed as the number of enzymes causing a change in absorbance per milligram of protein (U/mg protein). For each treatment, three enzyme assays were performed for each sample.

### 4.6. Statistical Analysis

The data were analyzed by one-way ANOVA with IBM SPSS 26.0, and the results of the Tukey test showed that the difference was statistically significant (*p* < 0.05). The results were expressed as means and standard deviations (SDs). The data statistics were performed by Microsoft Excel 2019, and the figure legends were drawn using Origin 2019b. The indexes were measured three times in parallel, and the results were averaged.

## 5. Conclusions

This study revealed the effects of different freezing treatments on the quality of sweet corn. After 42 days of storage, brine freezing and strong wind freezing greatly delayed the spoilage and browning of sweet corn, such as maintaining sweet corn color, moisture, hardness, soluble solids content, and antioxidant enzyme activity. In conclusion, brine freezing and strong wind freezing were the most suitable for freezing sweet corn and the most effective ways to extend storage time.

## Figures and Tables

**Table 1:** Effect of four different freezing methods on L*, a*, b* values of sweet corn. Values with different lowercase letters in the same sampling are significantly different (*p* < 0.05).

Sample	Time (Days)	L*	a*	b*
Fresh		75.71 ± 0.01 ^a^	4.98 ± 0.02 ^a^	46.22 ± 0.02 ^a^
Ultrasound-assisted freezing	7	71.95 ± 4.58 ^a^	4.41 ± 0.46 ^b^	45.21 ± 0.41 ^a^
	14	71.32 ± 2.28 ^a^	4.27 ± 0.94 ^b^	43.65 ± 2.67 ^ab^
	21	74.52 ± 2.88 ^a^	4.82 ± 0.39 ^ab^	43.15 ± 0.96 ^ab^
	28	71.94 ± 1.92 ^a^	5.21 ± 0.8 ^b^	42.33 ± 1.50 ^ab^
	35	69.76 ± 3.98 ^a^	5.35 ± 0.96 ^ab^	40.98 ± 2.32 ^b^
	42	69.23 ± 3.72 ^a^	5.59 ± 0.14 ^a^	40.32 ± 2.68 ^b^
Brine freezing	7	72.34 ± 4.48 ^a^	4.32 ± 0.87 ^b^	45.73 ± 1.20 ^b^
	14	71.63 ± 3.83 ^a^	4.16 ± 0.61 ^b^	44.72 ± 3.00 ^ab^
	21	73.86 ± 4.41 ^a^	4.69 ± 0.76 ^ab^	43.81 ± 2.42 ^ab^
	28	71.67 ± 3.17 ^a^	5.14 ± 0.81 ^b^	41.79 ± 3.22 ^ab^
	35	70.23 ± 2.05 ^a^	5.36 ± 0.72 ^ab^	41.02 ± 2.32 ^b^
	42	69.87 ± 2.23 ^a^	5.53 ± 0.41 ^a^	40.65 ± 0.62 ^b^
Strong wind freezing	7	73.11 ± 1.07 ^a^	4.52 ± 0.53 ^b^	46.62 ± 1.49 ^b^
	14	72.34 ± 3.52 ^a^	5.12 ± 0.55 ^a^	45.19 ± 0.77 ^ab^
	21	76.34 ± 3.60 ^a^	5.27 ± 0.43 ^a^	44.72 ± 2.38 ^ab^
	28	74.52 ± 3.45 ^a^	5.53 ± 0.28 ^a^	42.85 ± 0.81 ^ab^
	35	73.21 ± 4.16 ^a^	5.68 ± 0.63 ^a^	42.17 ± 1.88 ^b^
	42	71.23 ± 2.26 ^a^	5.71 ± 0.97 ^a^	41.61 ± 1.47 ^b^
Refrigerator freezing	7	70.21 ± 2.08 ^a^	5.17 ± 0.03 ^a^	43.09 ± 1.85 ^b^
	14	69.34 ± 1.91 ^a^	5.29 ± 0.98 ^a^	42.83 ± 1.38 ^ab^
	21	68.35 ± 1.68 ^a^	4.42 ± 0.67 ^b^	41.26 ± 2.30 ^ab^
	28	69.78 ± 4.94 ^a^	5.06 ± 0.34 ^b^	40.34 ± 3.20 ^ab^
	35	67.34 ± 0.81 ^a^	5.27 ± 0.56 ^a^	39.31 ± 3.69 ^ab^
	42	66.87 ± 4.38 ^a^	5.43 ± 0.25 ^a^	38.72 ± 0.01 ^b^

**Table 2:** Effect of different freezing methods on hardness, water loss, soluble sugar (c), and soluble solids content of sweet corn. Values with different lowercase letters in the same sampling are significantly different (*p* < 0.05).

Sample	Time (Days)	Hardness (g)	Weight Loss (%)	Soluble Sugar Content (mg/g)	Soluble Solids Content (%)
Fresh		1874.14 ± 184 ^a^	4.98 ± 0.02 ^a^	28.13 ± 3.92 ^a^	13.17 ± 0.93 ^a^
Ultrasound-assisted freezing	7	1325.43 ± 20.88 ^c^	10.3 ± 0.91 ^ab^	37.65 ± 2.41 ^ab^	10.90 ± 0.65 ^ab^
	14	1914.99 ± 35.37 ^b^	16.77 ± 2.47 ^a^	34.1 ± 3.61 ^a^	9.50 ± 0.57 ^a^
	2	2210.38 ± 27.21 ^a^	21.53 ± 3.55 ^ab^	29.22 ± 0.19 ^a^	8.77 ± 0.38 ^ab^
	28	2699.18 ± 36.56 ^b^	29.4 ± 2.78 ^a^	21.66 ± 2.84 ^a^	7.90 ± 0.54 ^a^
	35	3251.33 ± 54.77 ^c^	35.4 ± 3.93 ^a^	17.14 ± 1.68 ^a^	7.00 ± 0.08 ^a^
	42	3781.98 ± 42.2 ^b^	39.87 ± 5.21 ^a^	13.00 ± 1.46 ^a^	6.70 ± 0.59 ^a^
Brine freezing	7	1386.09 ± 8.25 ^b^	11.44 ± 1.28 ^ab^	38.21 ± 4.88 ^ab^	11.27 ± 0.95 ^ab^
	14	1799.81 ± 22.12 ^c^	19.33 ± 0.17 ^a^	33.72 ± 4.61 ^a^	9.17 ± 0.95 ^a^
	21	2064.84 ± 16.53 ^a^	22.7 ± 3.75 ^ab^	27.87 ± 1.82 ^a^	8.50 ± 0.16 ^ab^
	28	2768.49 ± 19.58 ^b^	31.67 ± 4.2 ^a^	20.12 ± 2.53 ^a^	8.13 ± 0.05 ^a^
	35	3388.90 ± 42.19 ^b^	32.33 ± 1.90 ^a^	16.43 ± 0.32 ^ab^	7.10 ± 0.73 ^a^
	42	3808.24 ± 40.97 ^b^	36.63 ± 4.96 ^a^	12.44 ± 1.50 ^a^	6.80 ± 0.67 ^a^
Strong wind freezing	7	1540.67 ± 3.86 ^a^	9.83 ± 0.42 ^b^	46.64 ± 2.42 ^a^	12.40 ± 0.36 ^a^
	14	1252.73 ± 2.27 ^d^	15.33 ± 1.17 ^a^	41.34 ± 4.08 ^a^	10.30 ± 0.50 ^a^
	21	1771.97 ± 7.93 ^a^	17.90 ± 3.20 ^b^	32.81 ± 1.70 ^a^	9.20 ± 0.45 ^a^
	28	2371.83 ± 39.2 ^c^	26.54 ± 2.21 ^a^	25.76 ± 1.53 ^a^	8.53 ± 0.68 ^a^
	35	3101.23 ± 58.27 ^d^	29.33 ± 3.97 ^a^	21.72 ± 2.32 ^a^	7.93 ± 0.05 ^a^
	42	3636.62 ± 55.44 ^c^	34.10 ± 1.42 ^a^	16.31 ± 1.55 ^a^	7.20 ± 0.71 ^a^
Refrigerator freezing	7	1056 ± 9.77 ^d^	13.23 ± 1.04 ^a^	33.23 ± 2.52 ^b^	10.27 ± 0.4 ^b^
	14	2033.5 ± 32.79 ^a^	19.47 ± 1.44 ^a^	30.32 ± 4.26 ^a^	8.40 ± 0.99 ^a^
	21	2490.63 ± 37.7 ^a^	29.37 ± 0.63 ^a^	21.12 ± 2.31 ^b^	7.90 ± 0.14 ^b^
	28	3036.62 ± 29.63 ^a^	33.13 ± 2.53 ^a^	14.16 ± 0.49 ^b^	7.30 ± 0.22 ^a^
	35	3619.79 ± 68.33 ^a^	34.40 ± 5.46 ^a^	10.63 ± 1.95 ^b^	6.8 ± 0.45 ^a^
	42	4081.56 ± 57.37 ^a^	37.87 ± 3.42 ^a^	7.75 ± 1.20 ^b^	6.10 ± 0.29 ^a^

**Table 3:** Sensory-evaluation project.

	Tissue	Smell	Appearance
10	Solid texture	Refreshing fragrance	The grains are full and arranged neatly
8	There is no shrinkage	No peculiar smell	Some grains are not full
6	Slight atrophy	No fragrance, slightly peculiar	Slightly poor color
4	Obvious atrophy but not serious	Obvious odor, but not serious	The corn kernels began to show individual mildew
2	Seriously atrophy	Severe odor	A large area of corn mildew occurred
0	All severely atrophied and moldy	Stench	Sweet corn rotted

## Data Availability

All data are contained within the manuscript and can be shared upon request to the corresponding author.
